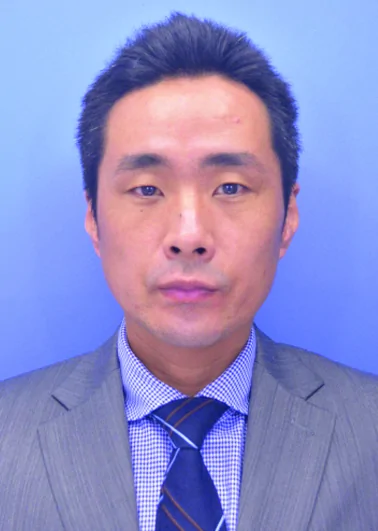# Deuterated Alkyl Sulfonium Salt Reagents; Importance of H/D Exchange Methods in Drug Discovery

**DOI:** 10.1002/cmdc.202400201

**Published:** 2024-06-25

**Authors:** Riku Ogasahara, Kazuho Ban, Miyu Mae, Shuji Akai, Yoshinari Sawama

**Affiliations:** ^1^ Graduate School of Pharmaceutical Sciences Osaka University 1-6 Yamada-oka Suita Osaka 565-0871 Japan; ^2^ Deuterium Science Research Unit Center for the Promotion of Interdisciplinary Education and Research Kyoto University Yoshida, Sakyo-ku Kyoto 606-8501 Japan

**Keywords:** Deuteration, Alkylation, Sulfonium salt, Heavy drugs, Kinetic isotope effect

## Abstract

Deuterated drugs (heavy drugs) have recently been spotlighted as a new modality for small‐molecule drugs because the pharmacokinetics of pharmaceutical drugs can be enhanced by replacing C−H bonds with more stable C−D bonds at metabolic positions. Therefore, deuteration methods for drug candidates are a hot topic in medicinal chemistry. Among them, the H/D exchange reaction (direct transformation of C−H bonds to C−D bonds) is a useful and straightforward method for creating novel deuterated target molecules, and over 20 reviews on the synthetic methods related to H/D exchange reactions have been published in recent years. Although various deuterated drug candidates undergo clinical trials, approved deuterated drugs possess CD_3_ groups in the same molecule. However, less diversification, except for the CD_3_ group, is a problem for future medicinal chemistry. Recently, we developed various deuterated alkyl (*d*_n_‐alkyl) sulfonium salts based on the H/D exchange reaction of the corresponding hydrogen form using D_2_O as an inexpensive deuterium source to introduce CD_3_, CH_3_CD_2_, and ArCH_2_CD_2_ groups into drug candidates. This concept summarises recent reviews related to H/D exchange reactions and novel reagents that introduce the CD_3_ group, and our newly developed electrophilic *d*_n_‐alkyl reagents are discussed.

## Graphical abstract

Recently, deuterated drugs have been highlighted as a new modality, and H/D exchange reactions are powerful and straightforward synthetic tools. We have successfully established a novel concept to diversify deuterium drug discovery using *d*_n_‐alkylation reagents (*d*_n_‐alkyl diphenylsulfonium salts) with excellent D contents and perfect site selectivity, which are readily prepared by the H/D exchange reaction of the corresponding hydrogen form using inexpensive D_2_O.

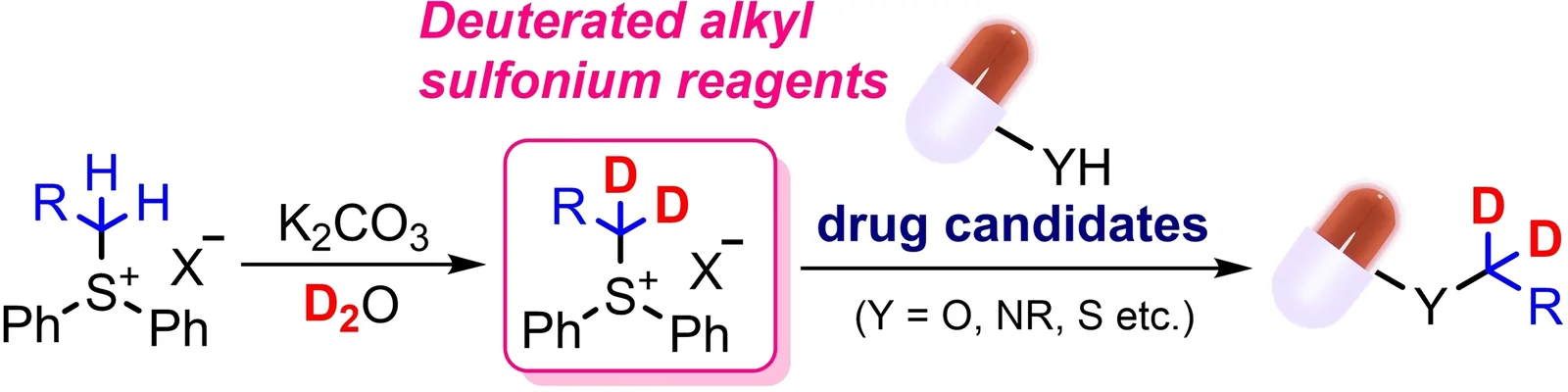

## Introduction

**Figure 1:**
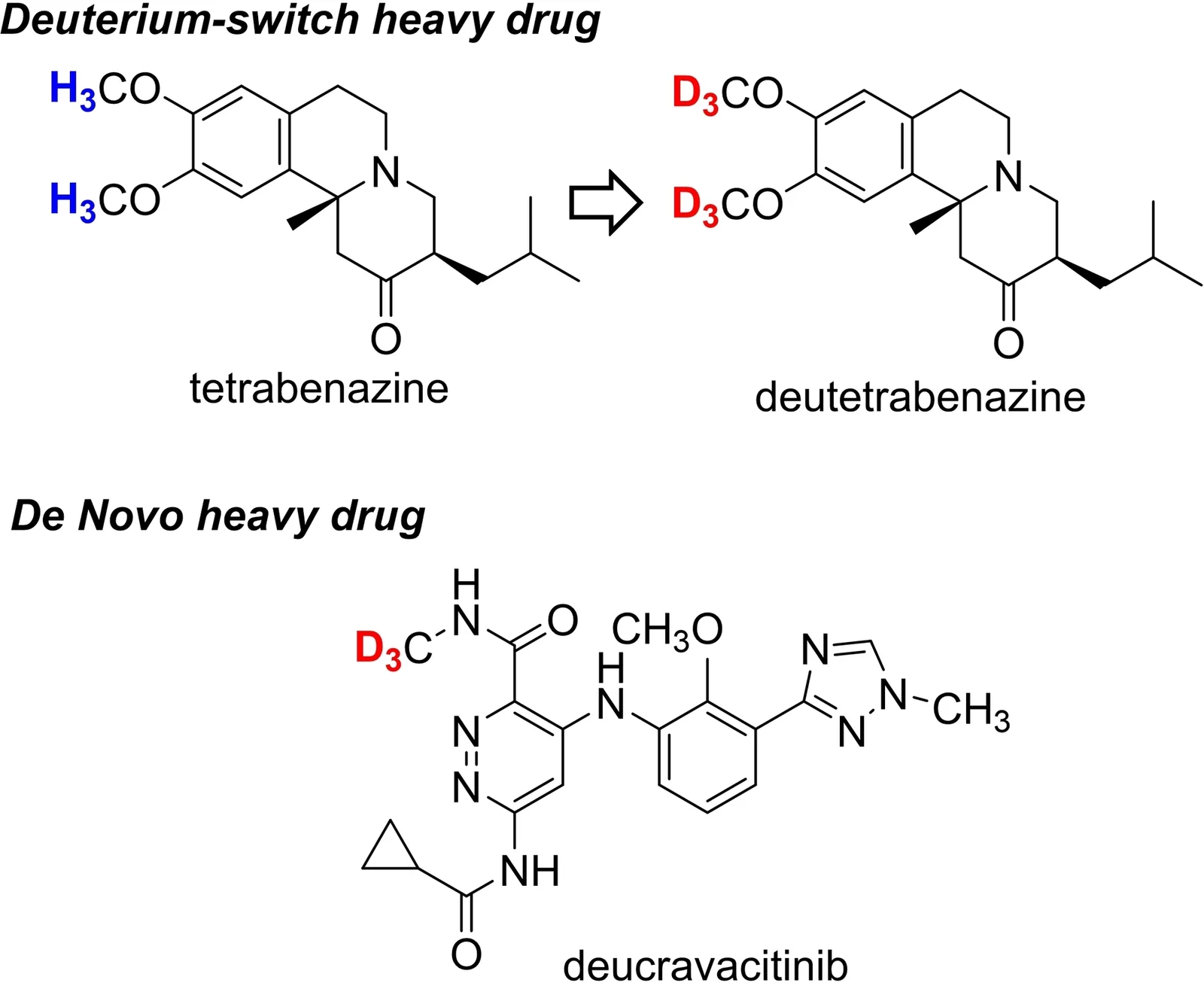
Approved heavy drugs.

Deuterium (D) is a nonradioactive and stable isotope of hydrogen (H). Compounds containing deuterium are widely used in various drug discoveries^[1]^ and studies to elucidate life phenomena (live‐cell Raman imaging,^[2]^ metabolic imaging using magnetic resonance imaging,^[3]^ and mass analysis).[^4^, ^5^] Among these, deuterated drugs (heavy drugs)^[1]^ have recently attracted considerable attention owing to the significant deuterium kinetic isotope effect (KIE) arising from the higher dissociation energy of the carbon−deuterium (C−D) bond compared with that of carbon−hydrogen (C−H) bond. Many pharmaceutical drugs undergo cytochrome P450 (CYP)‐mediated oxidative metabolism, and the replacement of C−H bonds with more stable C−D bonds at their metabolic sites, especially at the α‐position of heteroatoms, can improve their pharmacokinetics. For example, tetrabenazine (Figure 1; hydrogen form bearing no deuterium atom) is used to treat chorea in Huntington's disease, and its 9‐ and 10‐dimethoxy (OCH_3_) groups undergo CYP‐mediated metabolism to reduce its bioactivity. In contrast, the *deuterium‐switch* deutetrabenazine, possessing 9‐ and 10‐di‐(*d*_3_‐methoxy) (−OCD_3_) groups, acquires stronger metabolic tolerance and was approved as the first deuterated drug by the US Food and Drug Administration in 2017.^[1c]^ Furthermore, deucravacitinib, a *de novo* heavy drug, was approved for treating plaque psoriasis in 2022.^[1g]^

Considering deuterium‘s usefulness in various applications, including deuterated drugs, the development of various deuteration methods has recently gained momentum. Traditionally, deuterium introduction with skeleton transformation using reductants (LiAlD_4_/NaBD_4_/DCO_2_D) and reactive deuterium sources (CD_2_O/CD_3_I) has been adopted to construct deuterated drug candidates.^[6]^ In contrast, H/D exchange reactions (direct conversion of C−H bonds to C−D bonds) can be powerful tools for targeting deuterated molecules. Various H/D exchange methods using acids, bases, metal catalysts, or photocatalysts have been developed and summarized in review articles (Table 1).[^7^, ^8^, ^9^, ^10^, ^11^, ^12^, ^13^, ^14^, ^15^, ^16^, ^17^, ^18^, ^19^, ^20^, ^21^, ^22^, ^23^, ^24^, ^25^, ^26^] D_2_O, CD_3_OD, DMSO‐*d*_6_, D_2_, C_6_D_6_ etc., were utilized as the deuterium sources in each reaction, and using D_2_O as an abundant and inexpensive deuterium source was considered ideal.

**Table 1:** Reviews for H/D exchange methods in 2015–2024.

Reference	Year	**method (catalysts and reagents)**	**representative targets**
[7]	2015	acids.	aromatic moiety of lignans.
[8]	2018	metal oxides, metal films.	alkanes.
[9]	2018	acids/bases, Pt, Ru, Pd, Rh, Ir, and Fe catalysts.	aromatics, alkenes, alkyl amines, alkyl alcohols, amino acids, olefins, hydrosilanes, hydroborans.
[10]	2018	platinum group metal on carbons.	aromatics, benzylic positions, alkanes, sugars, saturated fatty acids
[11]	2019	Fe, Ni, and Co metals.	aromatics, benzylic positions.
[12]	2019	Ru, Ni, Mo, Mn, Fe, Ir, Pt, and base catalysts.	adjacent to heteroatoms (O, N, and S).
[13]^[a]^	2020	photocatalysts (Ir catalyst, 4CzIPN, TBADT and acridinium salt).	alkyl amines, hydrosilanes, aldehydes.
[14]	2020	acids/bases, Pd. Pt, Ru, Ir, Co, Mn, and Fe catalysts, photocatalyst (4CzIPN).	aromatics, benzylic positions, alkanes, alkyl amines, alkyl alcohol, sugars, amino acids, peptides, alkyl sulfides.
[15]	2020	Ir, Ru, Rh, Pt, and Pd catalysts.	aromatics, olefins, terminal alkynes.
[16]	2021	Ru, and Ir catalysts.	alkyl amines, aromatics, nucleotides, amino acids, peptides, alkyl sulfides, alkyl alcohols.
[17]^[a]^	2021	photocatalysts (4CzIPN, Ir catalysts, acridinium salt, and TBADT).	alkyl amines, amino acids, peptides, aldehydes, hydrosilanes.
[18]	2021	bases/acids, Ru, Ni, Pd, Ir, and Pt catalysts.	alkyl ketone, amino acids, peptides, alkyl alcohols, sugars, aromatics, benzylic positions, alkyl amines, alkyl sulfides.
[19]^[a]^	2021	Ru, Ir, and Pt catalysts, acids/bases.	alkyl cyanide, alkyl ketones, terminal alkyne, aromatics, alkyl alcohols, olefins, hydrosilanes.
[20]^[a]^	2022	photocatalysts (TBADT, 4CzIPN and Ir catalyst).	alkyl amines, peptides, aldehydes, hydrosilanes.
[21]^[a]^	2022	Ir, and Ru catalysts, photocatalysts (TBADT and 4CzIPN), NHCs.	aldehydes.
[22]^[a]^	2022	NHCs, photocatalysts (TBADT and 4CzIPN), Ir, Ru catalyst	aldehydes.
[6]^[a]^	2022	Pd, Pt, Rh, Ru, Ir, Ni, Mn, Co, Fe, and Ag catalysts, photocatalysts (4CzIPN, and Ir catalysts), acids/bases, NHCs.	aromatics, olefins, terminal alkynes, alkyl alcohols, alkyl sulfides, alkyl amines, amino acids, alkyl cyanides, allylic positions, benzylic positions, aldehydes, carbonyl (α position).
[23]^[a]^	2022	Ir, Pd, Rh, Ru, Ni, Cu, Fe, Ag, Co, and Mn catalysts, acids/bases, NHCs, AIBN, photocatalysts (4CzIPN, Ir catalyst, acridinium salt, and TBADT).	aromatics, olefins, terminal alkynes, alkyl amines, alkyl amides, amino acids, peptide, alkyl sulfides, benzylic positions, alkyl alcohols, nitromethane, saturated fatty acids, sugars, nucleotides (aromatics), aldehydes, carbonyl (α position).
[24]	2023	bases.	aromatics, benzylic positions, olefins, anisole, *Si‐*CH_3_.
[25]^[a]^	2023	acids/bases, Fe, Co, Rh, and Ru catalysts, photocatalyst (Ir catalyst), protein.	aromatics, olefins, benzylic positions, amino acids, aliphatic carboxylic acid, NHCs.
[26]^[a]^	2024	Ru, and Ir catalysts, photocatalyst (3DPA2FBN and 4CzIPN), AIBN.	alkyl amines, aromatics, amino acids.

[a] Other deuteration methods were also described, such as hydrogen/halogen exchange, reductive deuteration, deuteration via radical reaction intermediates, etc.; 4CzIPN: 1,2,3,5‐Tetrakis(carbazol‐9‐yl)‐4,6‐dicyanobenzene, TBADT: tetrabutylammonium decatungstate, NHCs; *N*‐heterocyclic carbenes, AIBN; azobis(isobutyronitrile), 3DPA2FBN; 2,4,6‐Tris(diphenylamino)‐3,5‐difluorobenzonitrile.

Although various deuterated drug candidates are undergoing clinical trials,[^1f^, ^1i^] approved deuterated drugs till now possess CD_3_ groups in the same molecule (Figure 1). These compounds were synthesized from commercially available CD_3_OD, CD_3_I, (CD_3_)_2_SO_4_, CD_3_NH_2_ and so on. Additionally, recently developed CD_3_ (*d*_3_‐methyl)‐linked sulfonium salts are useful tools for introducing CD_3_ groups into drug candidates. However, the high costs derived from commercially available deuterated reagents and less diversification, except for the CD_3_ group, are problematic for future medicinal chemistry. Recently, we developed various deuterated alkyl (*d*_n_‐alkyl) sulfonium salts based on the H/D exchange reaction of the corresponding hydrogen form using D_2_O as an inexpensive deuterium source to introduce CD_3_, CH_3_CD_2_, and ArCH_2_CD_2_ groups into drug candidates. Therefore, these novel introduction methods for the CD_3_ group were considered in detail, and the utility of the developed deuterated alkyl (*d*_n_‐alkyl) sulfonium salts is described below.

## d_3_‐Methyl Sulfonium Salt Reagents[^27^, ^28^]

**Figure 2:**
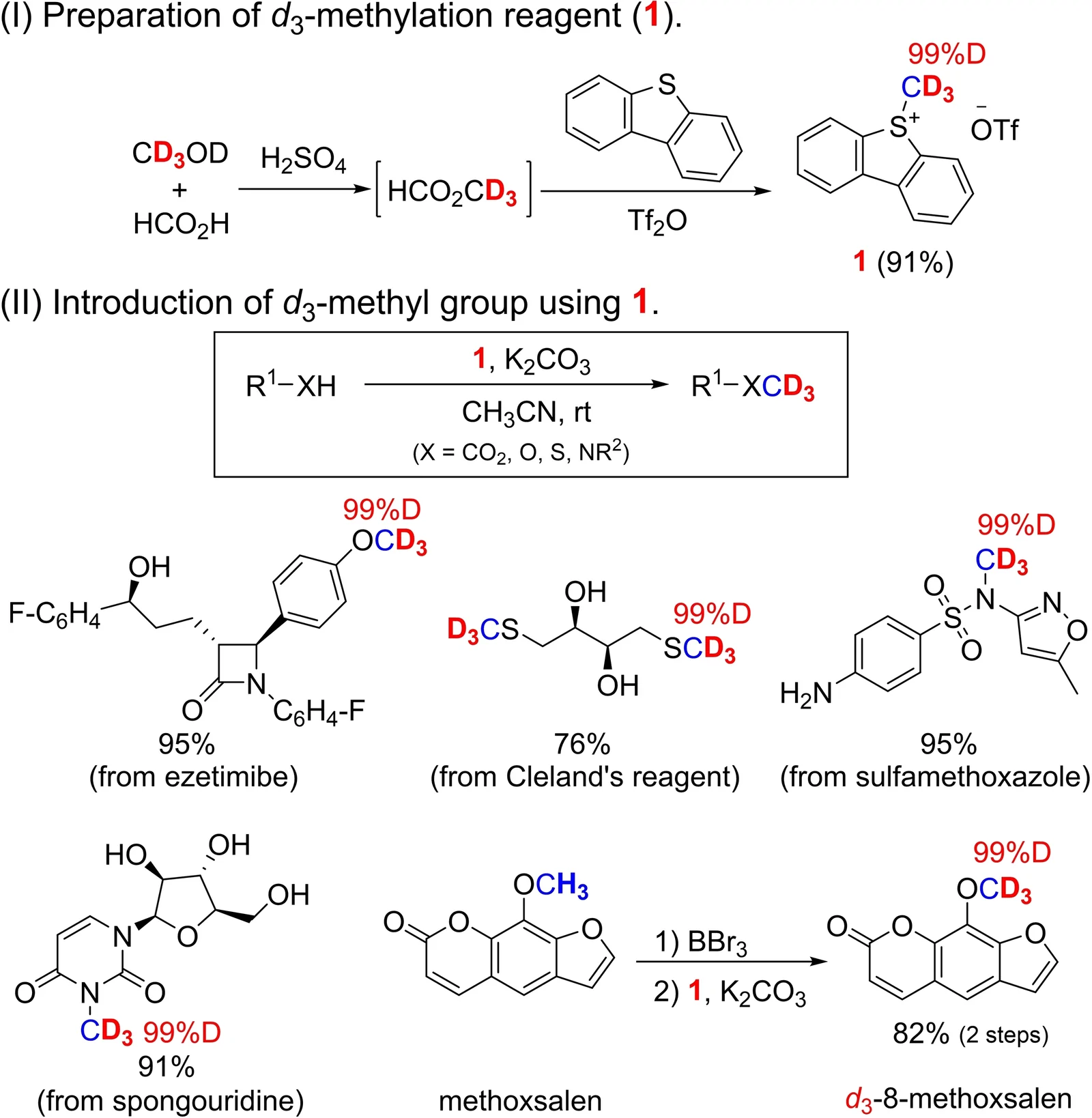
*d*_3_‐Methyl dibenzothiophenium salt reagent (Wang and Zhao's method). Tf_2_O; trifluoromethanesulfonic anhydride.

Wang and Zhao developed an electrophilic *d*_3_‐methylation reagent (**1**) from CD_3_OD and dibenzothiophene (Figure 2‐I).^[27]^ The reaction of CD_3_OD with HCO_2_H under acidic conditions gave *d*_3_‐methyl formate (HCO_2_CD_3_), which was then treated with dibenzothiophene and trifluoromethanesulfonic anhydride (Tf_2_O) to afford *d*_3_‐methyl dibenzothiophenium salt (**1**) as *d*_3_‐methylation reagent with 99 %D contents. **1** was efficiently reacted with various nucleophilic heteroatoms within the pharmaceutical drugs (ezetimibe, sulfamethoxazole, and spongouridine) and their related compounds (Cleland's reagent) to form the corresponding *d*_3_‐methylated products without any loss of D contents (Figure 2‐II). Additionally, the substitution switch from the CH_3_ group of methoxsalen to the CD_3_ group was accomplished by demethylation using BBr_3_ and subsequent *d*_3_‐methylation using **1**.

**Figure 3:**
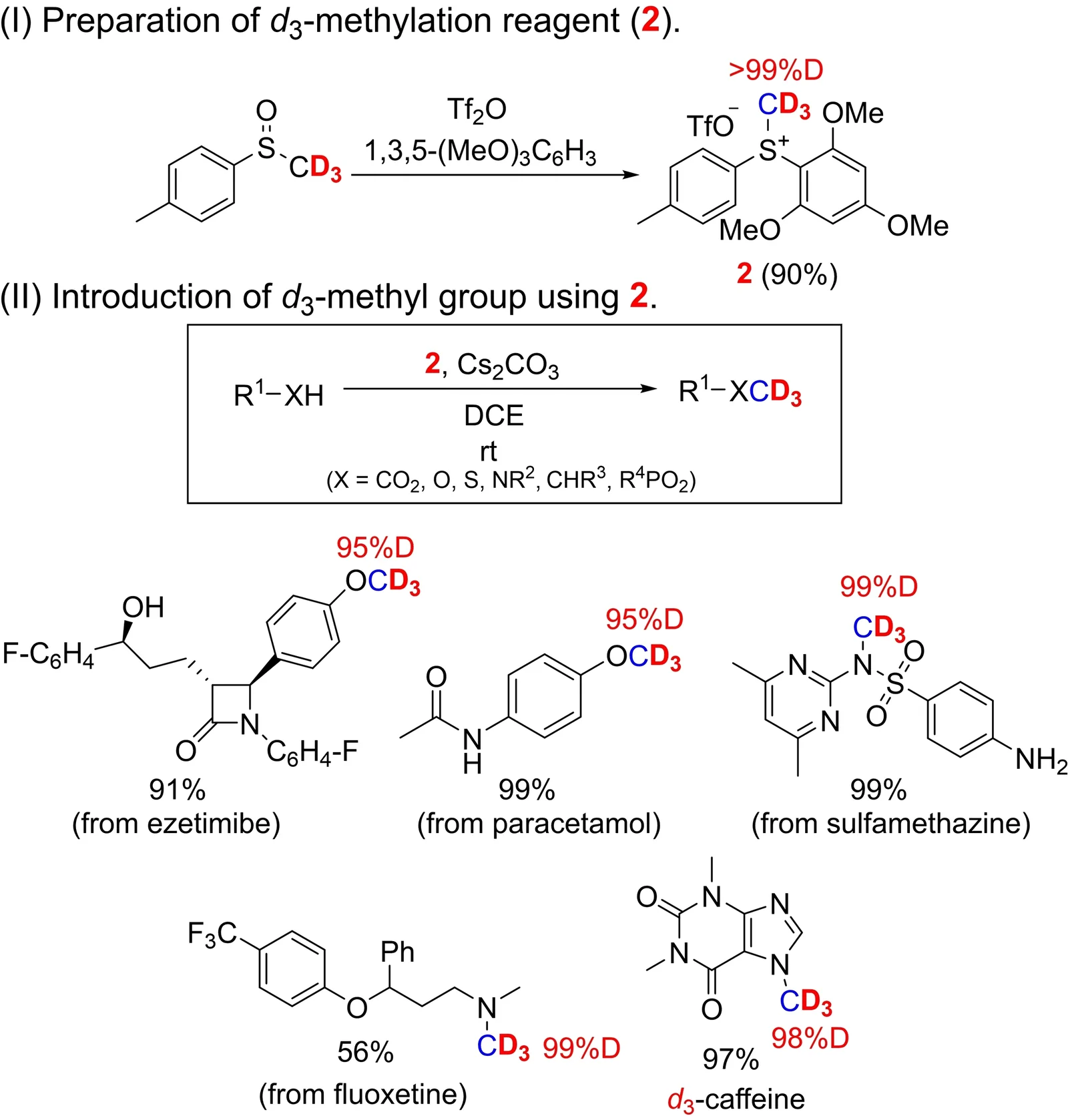
*d*_3_‐Methyl bis‐aryl sulfonium salt reagent (Meng and Tan′s method). DCE; 1,2‐dichloroethane.

Meng and Tan also developed a *d*_3_‐methylation reagent (**2**) consisting of a diaryl sulfonium salt (Figure 3‐I).^[28]^ **2** was prepared from the corresponding *d*_3_‐methyl *p*‐methyl(phenyl) sulfoxide using Tf_2_O and electron‐sufficient 1,3,5‐trimethoxybenzene. Although the preparation method for *d*_3_‐methyl *p*‐methyl(phenyl) sulfoxide has not been described in the literature, it can be assumed to be constructed by *d*_3_‐methylation of *p*‐methylbenzenethiol using CD_3_I and subsequent oxidation of the sulfur atom. Furthermore, *d*_3_‐methylated products were synthesized from ezetimibe, paracetamol, sulfamethazine, and fluoxetine using **2** as the electrophilic *d*_3_‐methylation reagent, and *d*_3_‐caffeine was also prepared. Although the products possessed outstanding D contents, a slight loss of D contents arising from an undesirable D/H exchange reaction during the *d*_3_‐methylation process was observed in some cases.

These two methods efficiently introduce *d*_3_‐methyl group into pharmaceutical drugs and bioactive compounds. However, expensive CD_3_ sources, such as CD_3_OD and CD_3_I, are required. Therefore, it is crucial to prepare a CD_3_ source from inexpensive and abundant D_2_O by the H/D exchange reaction and diversify the deuterated alkyl reagents, enabling the introduction of a limited number of deuterium atoms at the required positions (neighbouring positions at heteroatom; CYP‐mediated metabolic positions).

## Deuterated Alkyl Sulfonium Salt Reagents (Our Work)^[29]^

**Figure 4:**
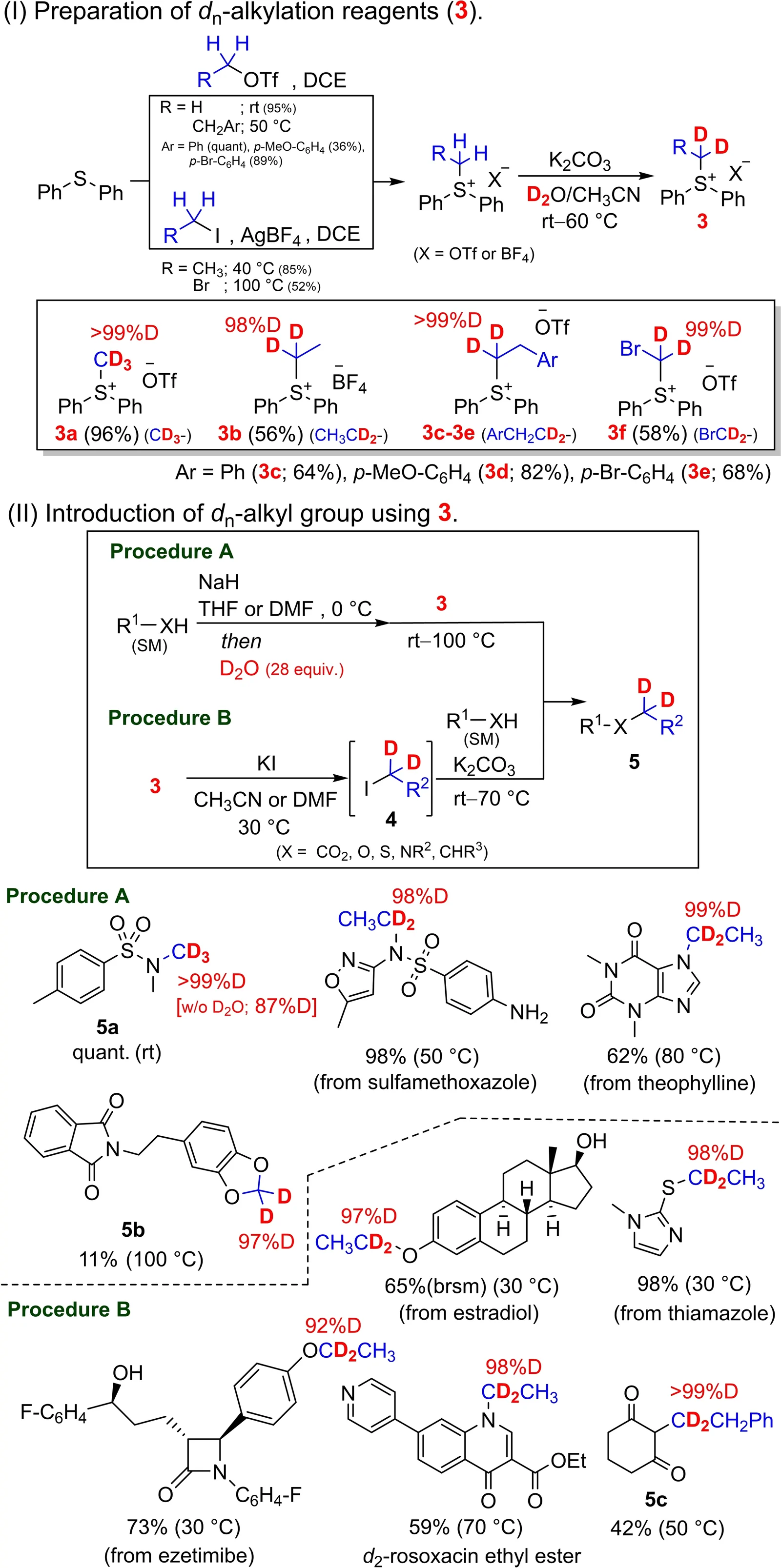
Deuterated alkyl diphenylsulfonium salt reagents. DMF; *N*,*N*‐dimethylformamide, THF; tetrahydrofuran, SM; starting material, brsm; based on recovered starting material.

Recently, we developed various deuterated alkyl (*d*_n_‐alkyl) diphenylsulfonium salts (**3**) based on the H/D exchange reaction of the corresponding hydrogen forms using D_2_O as an inexpensive deuterium source, to introduce *d*_n_‐alkyl moieties into drug candidates (Figure 4).^[29]^ Hydrogen forms (alkyl sulfonium salts) were easily prepared by the coupling of diphenylsulfide with alkyl triflate or alkyl iodide, and converted to **3** with excellent D contents and perfect site‐selectivity under basic conditions using K_2_CO_3_ and D_2_O (Figure 4‐I; alkyl dibenzosulfonium salt, such as **1** in Figure 2, was an inadequate substrate, and underwent hydrolysis to decompose the structure). Consequently, *d*_3_‐methyl‐(**3 a**) as well as *d*_2_‐ethyl‐(**3 b**), *d*_2_‐phenethyl‐(**3 c**), *d*_2_‐*p*‐methoxyphenethyl‐(**3 d**), *d*_2_‐*p*‐bromophenethyl‐(**3 e**), and *d*_2_‐bromomethyl‐(**3 f**) substituted sulfonium salts were successfully prepared. **3** was then utilized as an electrophilic *d*_n_‐alkylating reagent (**3**) for various heteroatom‐containing nucleophiles and 1,3‐diketone substrate under the two reaction conditions (Figure 4‐II, Procedures A and B). *N*‐methyl‐*N*‐*d*_3_‐methyl sulfonamide **5 a** with excellent D contents was obtained from *N*‐methylsulfonamide by successive addition of NaH, D_2_O, and **3 a** in THF (Procedure A: the reaction without D_2_O led to the loss of D contents during *d*_3_‐methylation step). Alternatively, *d*_n_‐alkyl iodide (**4**), prepared in situ from **3** and KI in CH_3_CN, was used as an electrophilic *d*_n_‐alkylating reagent (Procedure B). Consequently, *d*_3_‐methylation, *d*_2_‐ethylation, and *d*_2_‐phenethylation of complicated substrates including pharmaceuticals (sulfamethoxazole, theophylline, estradiol, thiamazole, ezetimibe, rosoxacin skeleton, etc.). Additionally, *d*_2_‐bromomethylated reagent **3 f** was reacted with a catechol moiety to give 1,2‐(*d*_2_‐methylene)dioxybenzene derivative (**5 b**).

**Figure 5:**
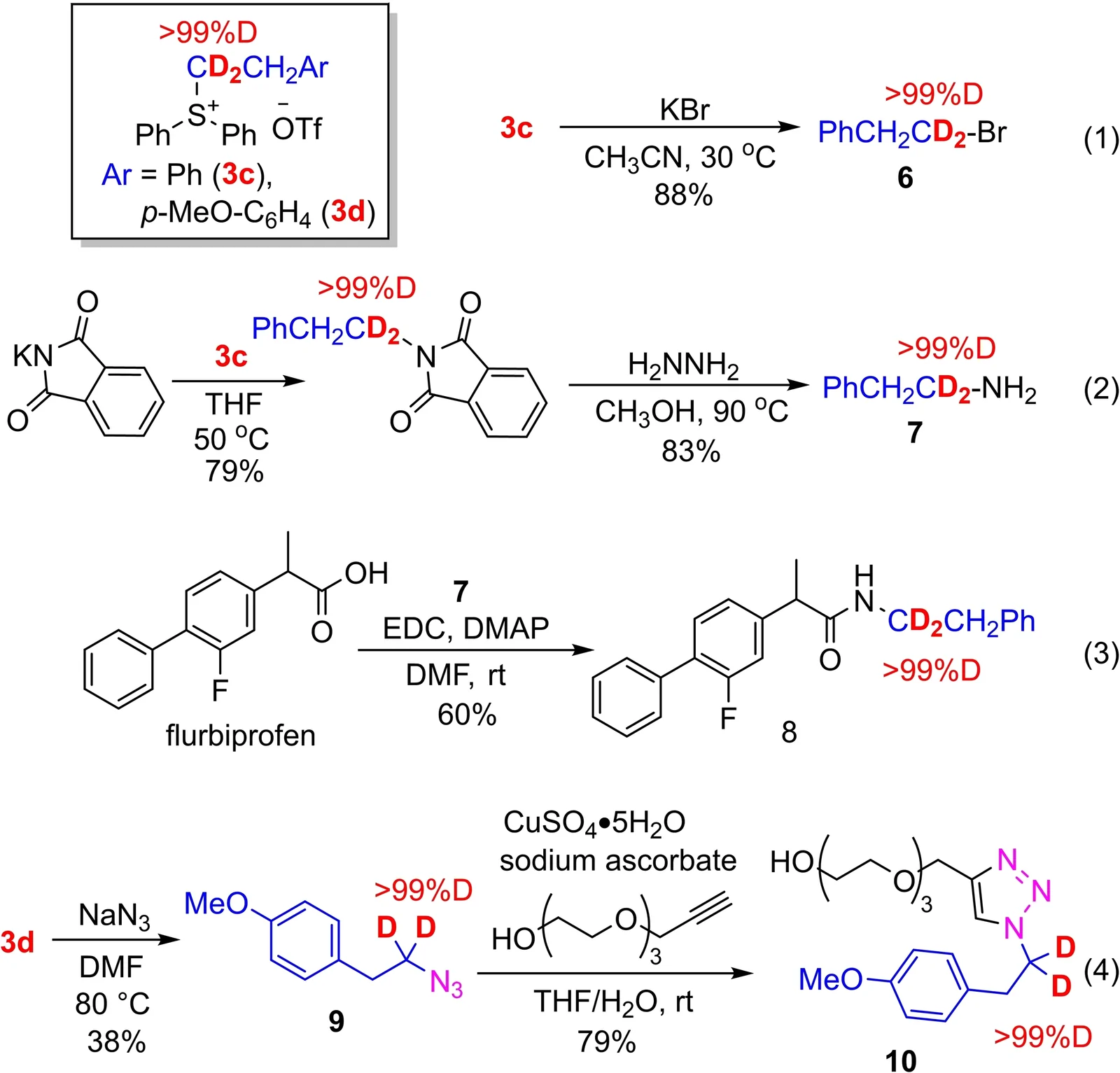
Further transformation of *d*_n_‐alkyl diphenylsulfonium salt reagents. EDC; 1‐(3‐dimethylaminopropyl)‐3‐ethylcarbodiimide Hydrochloride, DMAP; 4‐dimethylaminopyridine.

Moreover, **3** were converted into α‐deuterated alkyl halide/amine/azide compounds (Figure 5). 1‐*d*_2_‐Phenethyl bromide (**6**) with an excellent D contents was prepared from **3 c** and KBr (Figure 5‐1). 1‐*d*_2_‐Phenethylamine (**7**) was obtained through the Gabriel amine synthesis by coupling of potassium phthalimide with **3 c** and a subsequent reaction using hydrazine (Figure 5‐2). Furthermore, **7** underwent condensation with flurbiprofen to afford the amide product (**8**) without loss of D contents (Figure 5‐3). The reaction of **3 d** with NaN_3_ produced the azido product (**9**), which subsequently underwent Huisgen cyclization with *O*‐propargyl triethylene glycol to give a 2‐(4‐methoxyphenyl)(1‐*d*_2_‐ethyl)‐substituted triazole derivative (**10**; Figure 5‐4).

**Figure 6:**
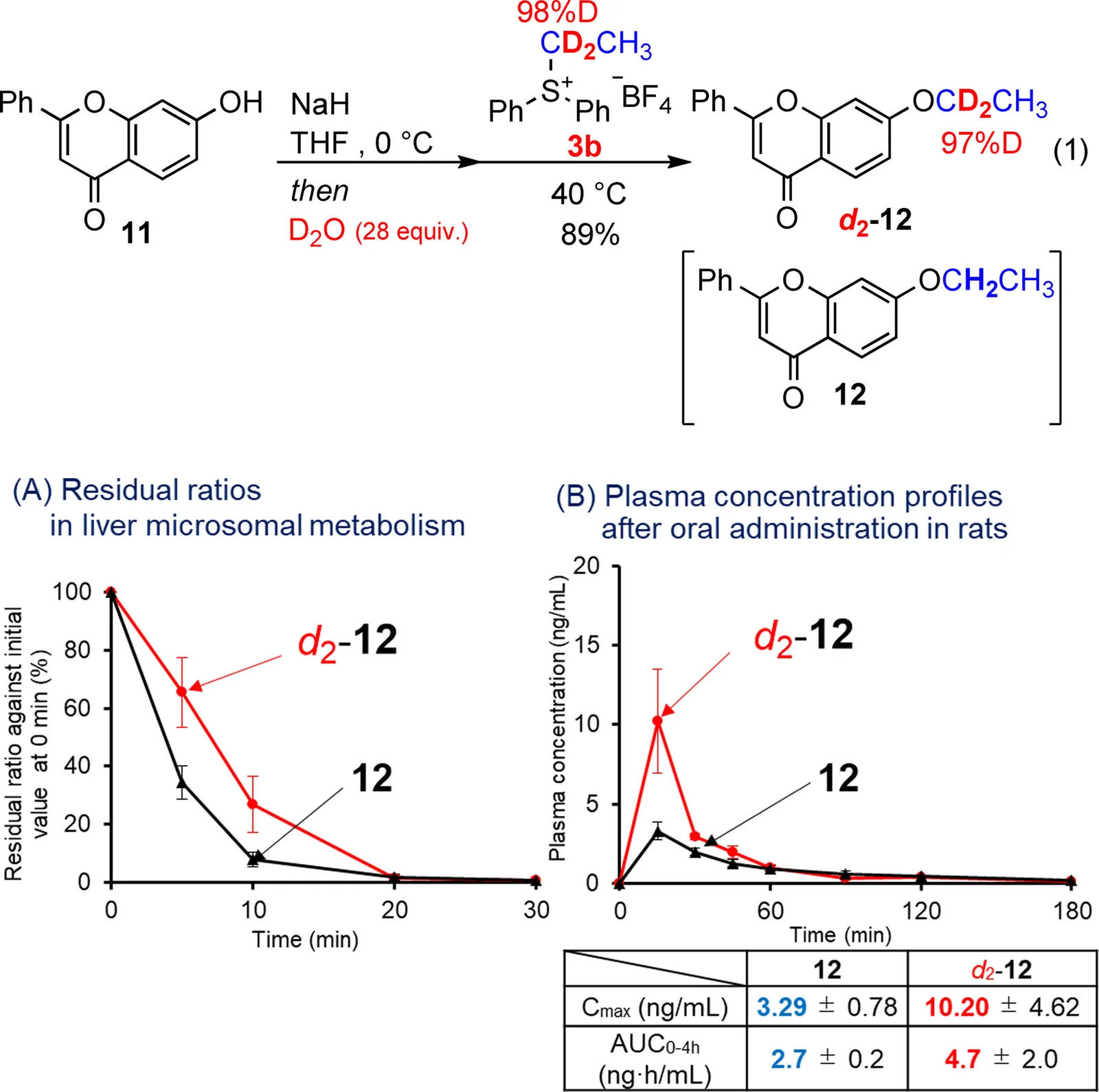
Synthesis of 7‐(*d*_2_‐ethoxy)flavone and KIE study. Cmax; maximum plasma concentration, AUC; area under curve

We performed a deuterium KIE study, relating to metabolic stability using 7‐(*d*_2_‐ethoxy)flavone (*d*_2_‐**12**), which was prepared from 7‐hydroxyflavone (**11**) and **3 b** according to Procedure A (Figure 4‐II) as a model antioxidant compound (Figure 6‐1). The residual ratios of *d*_2_‐**12** and the hydrogen form (7‐ethoxyflavone (**12**)) in the liver microsomal metabolism study revealed a significant effect of deuterium incorporation (Figure 6‐A). Additionally, the plasma concentration of *d*_2_‐**12** after oral administration to rats was higher than that of **12** (Figure 6‐B). This result indicated that both the C_max_ value and AUC were improved by deuterium KIE (parallel artificial membrane permeability assays showed that there were no significant differences in the membrane permeabilities of *d*_2_‐**12** and **12**). This concept is the first report on deuterium KIE using a deuterated ethyl moiety. Our strategy is regarded as an innovative drug discovery strategy.[^30^, ^31^]

## Conclusions and Outlook

The importance of introducing deuterium atoms into drug skeletons for drug discovery has recently been recognized worldwide. However, site‐selective deuteration with limited deuterium atoms remains a synthetic hurdle. H/D exchange reactions are powerful and straightforward tools, and using D_2_O as an abundant and inexpensive deuterium source is ideal. However, the site selectivity and D contents of the deuterated positions are generally problematic. We successfully established a novel concept to diversify deuterium drug discovery using *d*_n_‐alkylation reagents (*d*_n_‐alkyl diphenylsulfonium salt) with excellent D contents and perfect site selectivity, which were easily prepared by the H/D exchange reaction of the corresponding hydrogen form using D_2_O. Further improvements in diversification will continue to be investigated in our laboratory. This novel synthesis method is expected to contribute to the discovery of deuterium drugs in the future.

## Author Contributions

R.O. and K.B. equally contributed to arrange the contents.

## Conflict of Interests

The authors declare no conflict of interest.

## Biographical Information

*Yoshinari Sawama received a B.Sc. degree from the faculty of pharmaceutical sciences at Osaka University in 2001. He obtained his Ph.D. degree at Osaka University in 2006 under the supervision of Professor Yasuyuki Kita. After working as a postdoctoral fellow at Osaka University (2006–2007), Dortmund Technical University (2007–2009) and Ritsumeikan University (2009–2010), he was appointed as an Assistant Professor at Gifu Pharmaceutical University in 2010. He was promoted to Lecturer in 2015 and Associate Professor at the same university in 2017. In 2021, he moved to Osaka University as an Associate Professor*.